# Multicolor Quantum Dot Tracking Uncovers Phenotypic Rescue of DAT A559V Aberrant Diffusion Upon D2R Antagonism

**DOI:** 10.1021/acschemneuro.5c00897

**Published:** 2026-02-05

**Authors:** Ruben Torres, Oleg Kovtun, James R. McBride, Laurel G. Bellocchio, Sandra J. Rosenthal

**Affiliations:** † Department of Chemistry, 5718 Vanderbilt University, Nashville, Tennessee 37240, United States; ‡ Vanderbilt Institute of Chemical Biology, Vanderbilt University, Nashville, Tennessee 37240, United States; § Vanderbilt Institute for Nanoscale Science and Engineering, Vanderbilt University, Nashville, Tennessee 37240, United States; ∥ Department of Electrical and Computer Engineering, Vanderbilt University, Nashville, Tennessee 37240, United States; ⊥ Department of Pharmacology, Vanderbilt University, Nashville, Tennessee 37240, United States; # Department of Chemical and Biomolecular Engineering, Vanderbilt University, Nashville, Tennessee 37240, United States; ∇ Vanderbilt Interdisciplinary Materials Science Program, Vanderbilt University, Nashville, Tennessee 37240, United States

**Keywords:** quantum dot, two-color single particle tracking, dopamine transporter, dopamine receptor

## Abstract

The human dopamine transporter (DAT) is a presynaptic transmembrane protein that facilitates the reuptake of synaptically released dopamine. Several lines of evidence indicate that DAT dysfunction is linked to neuropsychiatric disorders. Moreover, the lateral membrane diffusion and clustering propensity of DAT are emergent properties that may factor into functional dopamine signaling. The disorder-associated DAT missense mutant A559V undergoes anomalous dopamine efflux (ADE) and increased lateral mobility and diffuse localization. The D2 dopamine autoreceptor short isoform (D2S), a popular antipsychotic target, signaling augments ADE in DAT A559V and may form stable DAT-D2S complexes. Using quantum dot (Qdot)-based single-molecule localization microscopy, we investigated the effect of D2S antagonism on DAT and DAT A559V membrane mobility in transfected HEK-293 cells. Single-color Qdot-DAT tracking shows phenotypic rescue of DAT A559V mobility upon D2S antagonism, while aberrant DAT A559V mobility is insensitive to ADE-linked CaMKII activity. Using two-color Qdot tracking of both the transporter and receptor, we report the first DAT-D2S colocalization lifetime in live cells. We show an increased propensity for both transporter types to colocalize with D2S, without impacting D2S diffusion speed under D2S antagonism. Downregulating D2S activity may stabilize DAT coconfinement in D2S microdomains on the cell surface.

## Graphical abstract

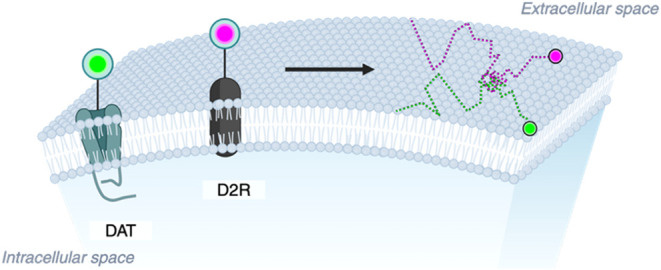

## Introduction

The catecholamine neurotransmitter dopamine is central to the neuronal pathways that control important behavioral roles, including reward, mood, and cognition. Considering that dopamine is critical to fundamental processes in mammalian brains, abnormal dopamine signaling has profound clinical consequences. It is linked to multiple neuropsychiatric brain disorders such as Parkinson’s disease, schizophrenia, major depression, bipolar disorder, autism spectrum disorder, and attention-deficit hyperactivity disorder.^1–4^ The presynaptic Na^+^/Cl^–^ coupled DAT determines dopaminergic signaling amplitude by shuttling receptor-available dopamine from the synaptic to the intracellular space. Importantly, genetic polymorphisms of the human DAT gene (*DAT1, SLC6A3*) have been associated with attention-deficit hyperactivity disorder, bipolar disorder, and juvenile dystonia.^1^ DAT trafficking at presynaptic termini is indisputably a major regulatory mode of synaptic strength in dopamine neurons. This process can be regarded as vertical trafficking, where DAT proteins are shuttled between the cell membrane and intracellular space. Consequently, exocytosis and endocytosis of DAT from vesicle pools contribute to the availability of functional DAT.^5^ An additional, underexplored layer of regulation involves the horizontal trafficking of DAT to presynaptic terminals by lateral membrane diffusion.

Interrogation of the lateral membrane diffusion of individual DAT proteins requires the use of DAT-specific nanometer-sized fluorophores. Inorganic semiconductor nanocrystals, colloquially known as quantum dots (Qdots), have been extensively used as fluorescent reporters^6^ to track several types of neurotransmitter receptors and transporter proteins (e.g., GABA_A_R, NMDAR, GlyR, SERT, DAT, D2R, D1R).^7–16^ Qdots consist of a CdSe core and a CdS shell and exhibit unique photophysical properties, making them an attractive probe for imaging applications. First, Qdots offer prolonged photostability for imaging acquisition times on the order of minutes to capture long-lived events. Additionally, Qdots have size-tunable narrow Gaussian photoluminescence profiles that permit simultaneous multicolor tracking with little to no spectral bleed through. Furthermore, as a product of high quantum yields and large absorption cross sections, Qdots are exceptionally bright upon laser irradiation, allowing high signal-to-noise ratios at low millisecond exposure times necessary for high frame rate acquisition. These unique properties have enabled the detection of single proteins in living cells targeted by antagonist- and antibody-conjugated biocompatible Qdots. Since DAT trafficking is a critical post-translational mechanism of synaptic regulation, our group pioneered the use of antagonist-conjugated Qdots to monitor individual membrane DAT proteins in live cells.^17,18^

Of particular importance is the DAT missense mutant A559V found in one female proband diagnosed with bipolar disorder, two brothers diagnosed with attention-deficit hyperactivity disorder, and two unrelated adolescent males diagnosed with autism spectrum disorder.^19–21^ Remarkably, this rare DAT variant displays normal dopamine uptake and vertical trafficking,^22,23^ yet bears a prominent outward flux of dopamine, anomalous dopamine efflux (ADE).^24,25^ Of several post-translational regulatory sites on the transporter, DAT A559V’s intracellular N-terminus is hyperphosphorylated, whereby inhibiting D2 dopamine receptor (D2R) and Ca^2+^/calmodulin-dependent protein kinase II (CaMKII) activity abolishes ADE in both HEK-293 cells expressing endogenous D2R and acute brain slices.^21,24–27^ Furthermore, phosphomimicked DAT N-terminus (S to D) shifts DAT to an efflux-prone state.^28^ We demonstrated that DAT A559V had increased membrane mobility and reduced clustering propensity sensitive to protein kinase C β (PKCβ) activity, while phosphomimicked N-terminus (S to D) DAT variants displayed DAT A559V-like diffusion patterns, supporting N-terminal hyperphosphorylation as a determinant of DAT membrane diffusion.^29^ Reports suggest that transient interactions between the DAT N-terminus and binding partners such as D2R intracellular loop 3 (ICL3) may lead to the formation of macromolecular complexes that are believed to be functional DAT units.^30,31^ Interestingly, D2R is an established drug target to treat Parkinson’s disease and schizophrenia.^32,33^ D2R exists in two canonical isoforms: short (D2S), believed to function as an autoreceptor on the presynaptic terminal along with DAT and long (D2L), believed to exist on the postsynaptic terminal.^34–37^ Here, we demonstrate the modulatory effects of D2S blockade on the spatiotemporal membrane diffusion patterns of DAT and its A559V variant. We show that the coding variant exhibits (i) phenotypic rescue of the lateral diffusion rate and confinement propensity upon D2S inhibition and (ii) insensitivity to ADE-linked CaMKII activity. Simultaneous two-color Qdot tracking of both the transporter and the receptor shows that (i) D2S diffusion is unresponsive to antagonism, (ii) D2S inhibition increases colocalization lifetime of D2S with both DAT and DAT A559V, and (iii) diffusional state switching to a less mobile state for both DAT types when colocalized with D2S. Our findings support the idea that DAT and D2S transiently coconfine into membrane domains through a DAT N-terminal phosphorylation-dependent manner.

## Results

### Phenotypic Rescue of DAT A559V Aberrant Membrane Diffusion upon D2R Inhibition

DAT A559V experiences faster and delocalized membrane or “lateral” trafficking linked to elevated N-terminal transporter phosphorylation levels,^29^ and ADE augmented by D2R signaling.^25^ To monitor the influence of D2R signaling on aberrant DAT A559V lateral trafficking, we implemented our DAT targeting strategy. Figure 1a illustrates the chemical structure of IDT444, the DAT-specific ligand used in our labeling architecture, furnished with a carbomethoxy-fluorophenyl cocaine analogue, an 11-carbon alkyl spacer providing necessary aliphaticity for the antagonist to reach its buried binding site, a PEG, and a biotin handle for affinity-based streptavidin-conjugated Qdot (SA-Qdot) binding. The A559V mutation is located on transmembrane domain 12 (Figure 1a). SAv-Qdot-IDT444 labeling has been successful in tracking single DAT proteins,^17,38–40^ specifically DAT A559V.^29^ HEK-293 cells were transiently transfected with either DAT or DAT A559V and were preincubated with the selective D2R inhibitor raclopride (RCP; 1 μM) 20 min^25,27^ prior to SAv-Qdot-DAT imaging using high-speed spinning disk confocal microscopy at 10 Hz. Representative reconstructed trajectories of DAT are shown in Figure 1b. IDT307 (4-(4-dimethylamino)­phenyl-1-methylpyridinium, APP+)^41,42^ was used to identify DAT-expressing cells. As a result of Qdot photostability, individual DAT pools persisting in the focal plane were captured up to the total duration of acquisition (600 frames, 100 ms exposure time; Figure 1c).

**Figure 1:**
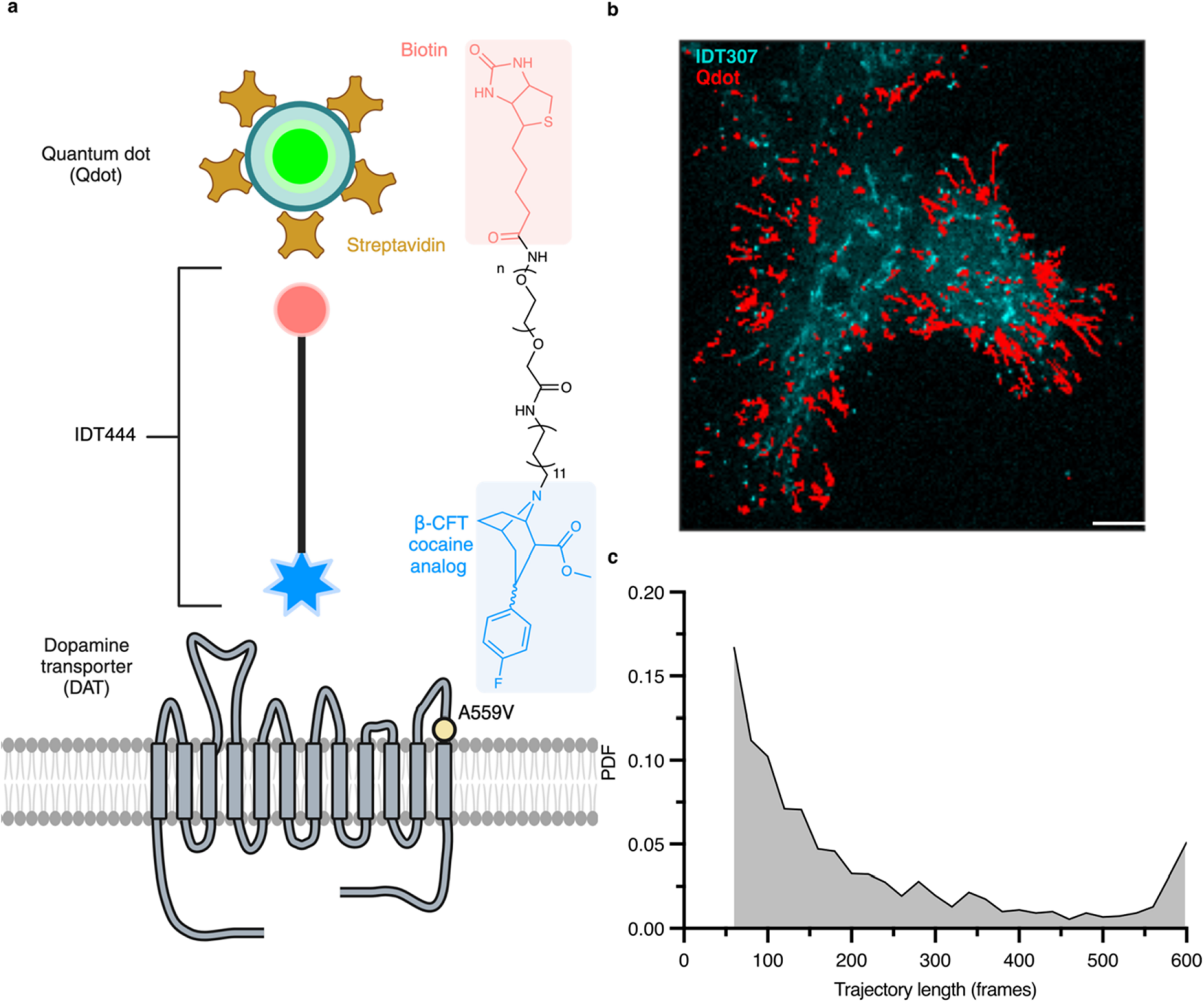
Single-color Qdot tracking method for DAT. (a) Cartoon outlining single Qdot-DAT labeling architecture and chemical structure of the DAT-specific IDT444 affinity tool and the location of A559V site highlighted in yellow (drawn not to scale). (b) Representative trajectories acquired over 60 s of Qdot-bound DAT superimposed to the IDT307 channel of a HEK-293 cell transiently expressing DAT taken on a spinning disk confocal microscope. Scale bar, 10 μm. (c) Representative probability density function (PDF) for DAT trajectory lengths sampled at a 100 ms frame rate (*n* = 2182 trajectories).

Mean jump distances (MJD) (i.e., the distance a single particle traveled between frames) were determined for populations of SAv-Qdot-labeled DAT and DAT A559V under basal conditions or RCP treatment (Figure 2a). No significant difference between all conditions was observed, and all peaks were centered around ∼80 nm. This MJD value aligns with the reported DAT MJD range.^43^ An instantaneous mobility parameter such as MJD alone does not accurately describe time-dependent diffusion dynamics,^44^ therefore, we determined the averaged mean square displacements (MSD) to describe the motion behavior for DAT and DAT A559V (Figure 2b). Both basal curves for DAT and DAT A559V show confined motion, and the DAT A559V slope is greater than that of DAT, consistent with previous reports.^29^ The slope for DAT A559V + RCP closely resembles those of DAT and DAT + RCP. Cumulative probability distributions (Figure 2c) of diffusion coefficients (*D*_MLE_) show a significant global reduction in DAT A559V diffusion rate upon D2R inhibition with RCP (*bottom*), and DAT is unaffected by D2R inhibition (*top*). The same trend is seen in Figure 2d with *D*_MLE_ distributions highlighting median values and interquartile ranges (25–75%) for each condition. In addition to diffusion coefficients, 5 s radial displacement vectors were calculated by obtaining particle distance and direction traveled in 5 s (50 frames at 10 Hz) normalized to the particle position at the first frame. Means of displacements for DAT are unaffected by D2R inhibition, but a significant reduction in displacement, identical to DAT displacement, is observed for DAT A559V upon D2R inhibition (Figure 2e).

**Figure 2:**
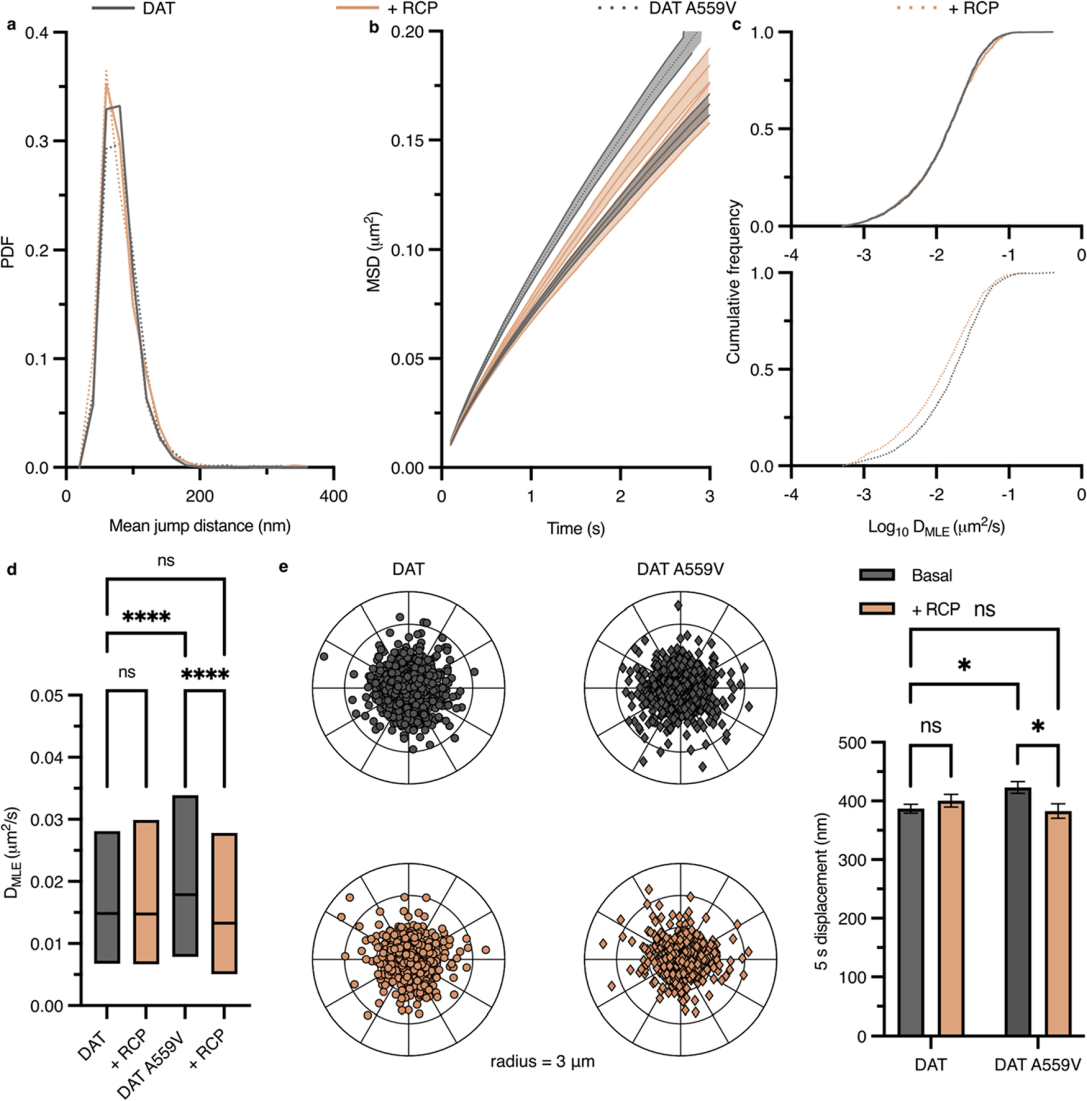
D2S inhibition elicits DAT A559V membrane diffusion recovery and has no effect on DAT in transiently transected HEK-293 cells. (a) PDF for total mean jump distances (MJD) for all conditions. (b) Average mean square displacement (MSD) plots for all conditions. Data are presented as mean ± SEM. (c) Cumulative frequency distribution of diffusion coefficients (*D*_MLE_) of DAT basal and RCP inhibition (*top*; Kolmogorov–Smirnov 2-sample test, *p* > 0.05) and DAT A559V basal and RCP inhibition (*bottom*; Kolmogorov–Smirnov 2-sample test, *p* < 0.0001). (d) Box plot showing the diffusion coefficient distributions in panel c. The median value is shown as the colored horizontal line in the box; the 25–75% IQR interval corresponds to the length of the colored box (Two-way ANOVA with Holm-Sidak multiple comparisons test). (e) Polar plots (*left*; outer radius limit = 3 μm) and 5 s displacement bar graph (*right*; Two-way ANOVA with Holm-Sidak multiple comparisons test). Displacements are normalized to their spatial origin. Data are presented as mean ± SEM *N* trajectories (DAT *n* = 2182; DAT + RCP, *n* = 1175; DAT A559V, *n* = 1544; DAT A559V + RCP, *n* = 990).

### Aberrant DAT A559V Membrane Diffusion is Insensitive to CaMKII N-Terminus Activity

Hyperphosphorylated DAT A559V experiences D2R-mediated ADE that is sustained by CaMKII phosphorylation of the DAT N-terminus, while inhibition of CaMKII or D2R reduces ADE.^25^ To assess whether D2R-mediated CaMKII activity linked to DAT ADE is coupled to aberrant DAT A559V lateral trafficking, the effects of general CaMKII inhibition by KN93 were explored. HEK-293 cells were transiently transfected with DAT A559V and green fluorescent protein (GFP)-tagged CaMKII wild-type or an inactive variant (K42R). The K42R mutation eliminates kinase activity^45–49^ and served to ablate low levels of endogenous CaMKII catalytic activity in a dominant negative manner. Cells were preincubated with 5 μM KN93 or 5 μM KN92, an inactive analogue, for 20 min prior to SAv-Qdot labeling. Figure 3a–d show representative trajectories from SAv-Qdot-DAT A559V cells coexpressing CaMKII or CaMKII K42R. CaMKII inhibition has no effect on DAT A559V MSD, diffusion rates, or 5 s displacements (Figure 3e–g). Similar null results were observed when endogenous CaMKII in HEK-293 cells was inhibited (Figure S1).

**Figure 3:**
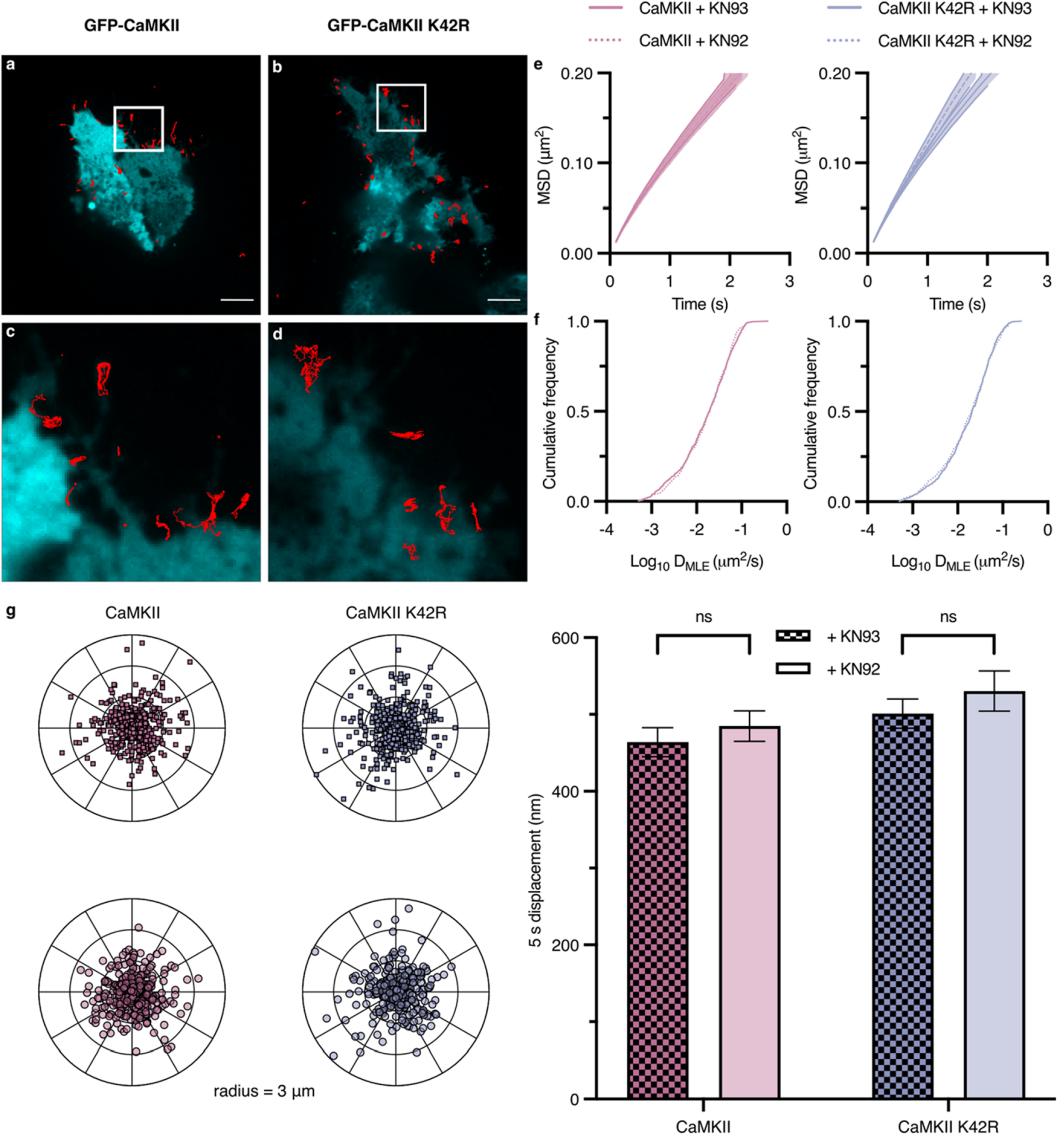
DAT A559V mobility is insensitive to CaMKII inhibition in transiently transfected HEK-293 cells. (a, b) Representative trajectories collected over 60 s of Qdot-bound DAT A559V coexpressed with either GFP-CaMKII or GFP-CaMKII K42R channel under CaMKII inhibition by KN93, taken on a spinning disk confocal microscope. Scale bar, 10 μm. (c, d) Images at 4× magnification of the images in a and b, respectively. (e) MSD plots of DAT A559V, CaMKII + KN93/KN92 (*left*) or DAT A559V, CaMKII K42R + KN93/KN92 (*right*). (f) Cumulative frequency distributions of diffusion coefficients (*D*_MLE_) of conditions from e (*left* and *right*; Kolmogorov–Smirnov 2-sample test, *p* > 0.05). (g) Polar plots (*left*; outer radius limit = 3 μm) and 5 s displacement bar graph (*right*; Two-way ANOVA with Holm-Sidak multiple comparisons test). Displacements are normalized to their spatial origin. Data are presented as mean ± SEM. N trajectories (CaMKII + KN93, *n* = 671; CaMKII + KN92, *n* = 507; CaMKII K42R + KN92, *n* = 642; CaMKII K42R + KN92, *n* = 445).

### Simultaneous Two-Color Qdot Imaging Probes DAT and D2S Interactions at High Spatiotemporal Resolution

Previous studies suggested the possibility of a functional transient DAT-D2S complex.^30,31,50,51^ To characterize the dynamic behavior of DAT and D2S, we leveraged Qdots once more to simultaneously monitor DAT and D2S on the membrane surface of live cells. Figure 4a shows our labeling approach to specifically label DAT and D2S. DAT and DAT A559V were labeled using the same approach shown in Figure 1a, and D2S was labeled using a primary/secondary antibody approach. A rabbit anti-D2S antibody raised against the extracellular D2S N-terminus (amino acids 11–26) was validated for D2S specificity (Figure S2) and used to label D2S. A goat antirabbit antibody Qdot 705 (Ab-Qdot 705) was attached to the primary antibody. In HEK-293 cells, D2R primarily resides in intracellular compartments.^27^ Therefore, we transiently transfected HEK-293 cells with DAT or DAT A559V along with untagged D2S to maximize the probability of observing transient interactions. Figure S3 shows that the diffusion coefficient and 5 s displacement trends observed in single-transfected HEK-293 cells (Figure 2) are conserved when the transporter and the receptor are coexpressed. A representative image of a HEK-293 cell labeled for both the transporter and receptor is shown in Figure 4b. DAT and D2S were imaged using dual-color total internal reflection fluorescence (TIRF) microscopy at 58 Hz. At 17 ms exposure times, clear spectral separation between SAv-Qdot 605 (DAT) and Ab-Qdot 705 (D2S) (3% estimated spectral overlap) and high signal-to-noise point spread functions were observed. Line scans of absolute grey value for each channel show Qdot emission peaks well above background noise (Figure 4c). Under these imaging parameters, Qdot fluorescence intermittency (blinking) was minimized, reducing the probability of long blinking events prematurely ending transient interactions (Figure S4). DAT and D2S trajectories were generated separately using the popular single particle analysis tool, TrackMate.^52,53^ To maximize spatiotemporal information from transporter and receptor trajectories, we implemented a trajectory analysis tool, ExTrack, developed by Simon et al.^54^ As a TrackMate extension, ExTrack generates global model parameters to calculate diffusion state probabilities at every time point and refine the positions of bound molecules. Using a two-state annotation model, Figure 4d (*left*) depicts a hypothetical trajectory where points that display diffusive motion are annotated with a higher probability of being in the diffusive state (Diff.; state 1) and points that display bounded motion are annotated with a higher probability of being in the immobilized state (Im.; state 0). The position refinement module (Figure 4d, *right*) increases the localization accuracy of bound positions. Due to conflicting reports on the existence of stable DAT-DS dimers^30,50,51,55^ and the experimental limitations of fluorescence microscopy, we instead described DAT-D2S interaction as diffraction-limited colocalization-codiffusion. Transient events in which surface DAT/DAT A559V and D2S colocalize (<1 pixel; 0.117 μm) were tracked over time and isolated from ExTrack-generated trajectories. Figure 4e demonstrates our approach to isolating colocalization events. A pair of colocalizing trajectories from Figure 4b was plotted over time, in which colocalized points were plotted with a black border. Time points where separation distances between proteins fell below the set threshold were considered to be colocalized. Our analysis can isolate multiple colocalization events within single trajectories.

**Figure 4:**
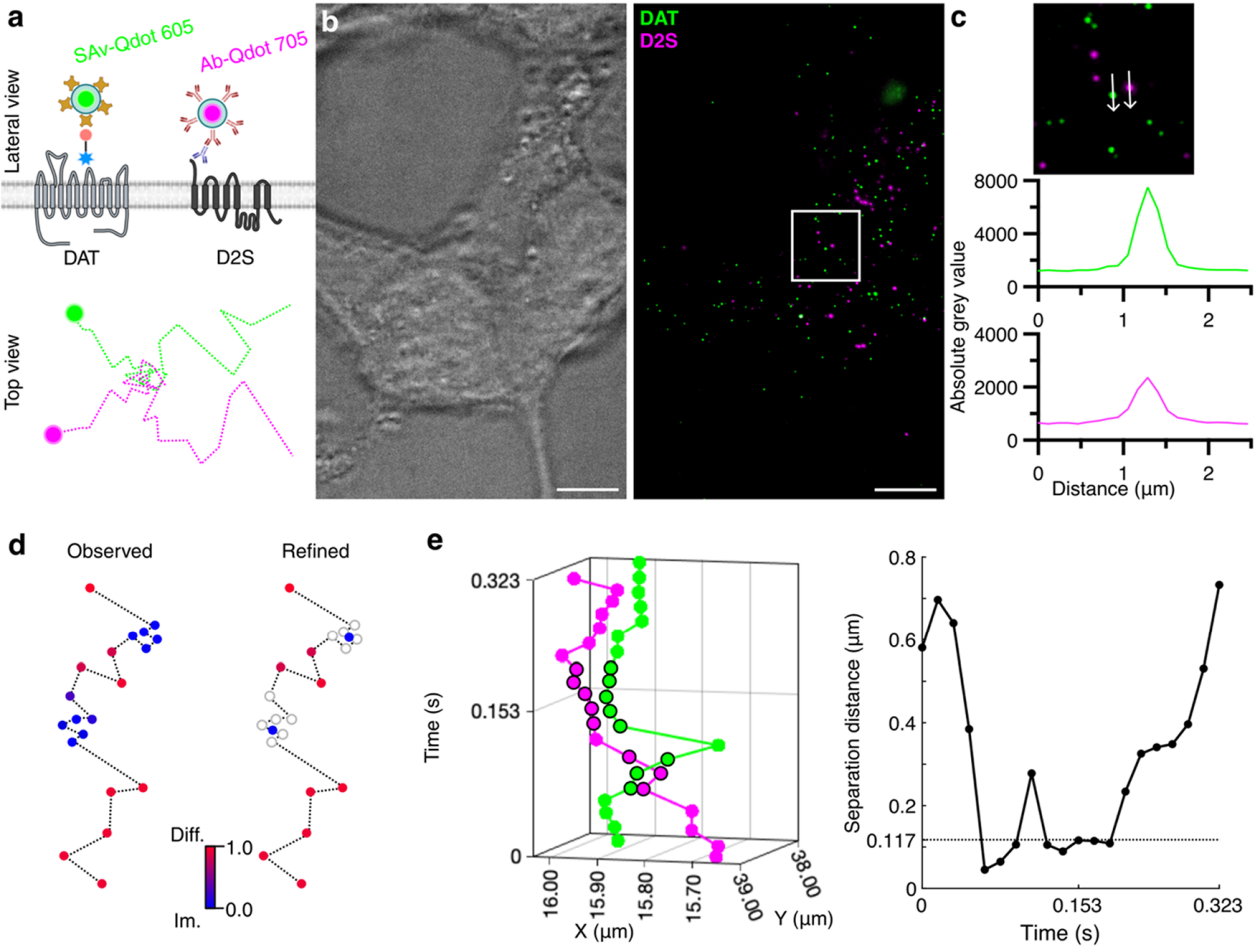
Two-color Qdot tracking and colocalization analysis principles for DAT and D2S. (a) Cartoon outlining antagonist-based Qdot-DAT and antibody-based Qdot-D2S surface labeling. Anti-D2S antibody recognizes the extracellular N-terminus of D2S. (b) Representative images of HEK-293 (brightfield) coexpressing DAT and D2S and corresponding Qdot fluorescence signal taken on a TIRF microscope at 17 ms frame rate. Scale bar, 10 μm. (c) 4× magnification (*top*) of b and line scans (*bottom*) of the absolute grey value for Qdot 605 and Qdot 705. (d) Cartoon depicting ExTrack diffusion state annotation (*left*) and position refinement (*right*) modules along a hypothetical trajectory. (e) Representative reconstructed trajectories of DAT and D2S surface diffusion, where instances of colocalization (1 pixel; 0.117 μm) are outlined in black (left) and separation distance plot between DAT and D2S over time (*right*; dotted line depicts colocalization distance threshold).

### D2S Mobility is Insensitive to RCP Inhibition

We used a two-state model to generate global diffusion parameters for each cell recorded under the outlined basal and RCP treatment. Under a two-state model assumption, a mobile population (1) and an immobile population (0) of proteins were determined. Strikingly, the trend in which DAT A559V diffusion speed (*D*_1_ = *D*_MLE_ for state 1) attenuation from RCP treatment and DAT’s insensitivity to RCP treatment was observed (Figure 5a). D2S *D*_1_ was unaffected by RCP treatment, regardless of coexpressed transporter type (Figure 5d). No change in *D*_0_ (*D*_MLE_ for state 0) was observed. This population of particles was interpreted as a mixture of both immobilized DAT/D2S and Qdots adhered to the glass coverslip (Figure 5b,e). A two-state analysis of SAv-Qdot 605’s adhered to the glass coverslip revealed *D*_0_ and *D*_1_ values (Figure S5) in agreement with prior reports for immobilized Qdots,^29,38,39^ supporting that Figure 5b,e *D*_0_ values more accurately represent immobilized DAT and D2S.^43,56–58^ Interestingly, the fraction of particles in mobile state 1 (F_1_) remained unchanged for both transporters and D2S (Figure 5c,f) especially considering that the diffusion speed for DAT A559V decreases under RCP treatment. The magnitude of all *D*_1_ and *D*_0_ diffusion coefficients were much larger than diffusion coefficients from single-color Qdot-DAT tracking, likely to be an outcome of video acquisition sampling rate.^59^ In a previous report, a significant difference (28%) in median *D*_MLE_ values for fluorescent protein-tagged DAT was observed when the sampling rate was increased from 50 to 20 ms exposure time.^40^ The difference between DAT and D2S D_1_ values was to be expected. D2S is smaller in size than DAT, and the untethered or unconstrained receptor would be expected to diffuse on the membrane surface faster than DAT (Figure 5a,d).

**Figure 5:**
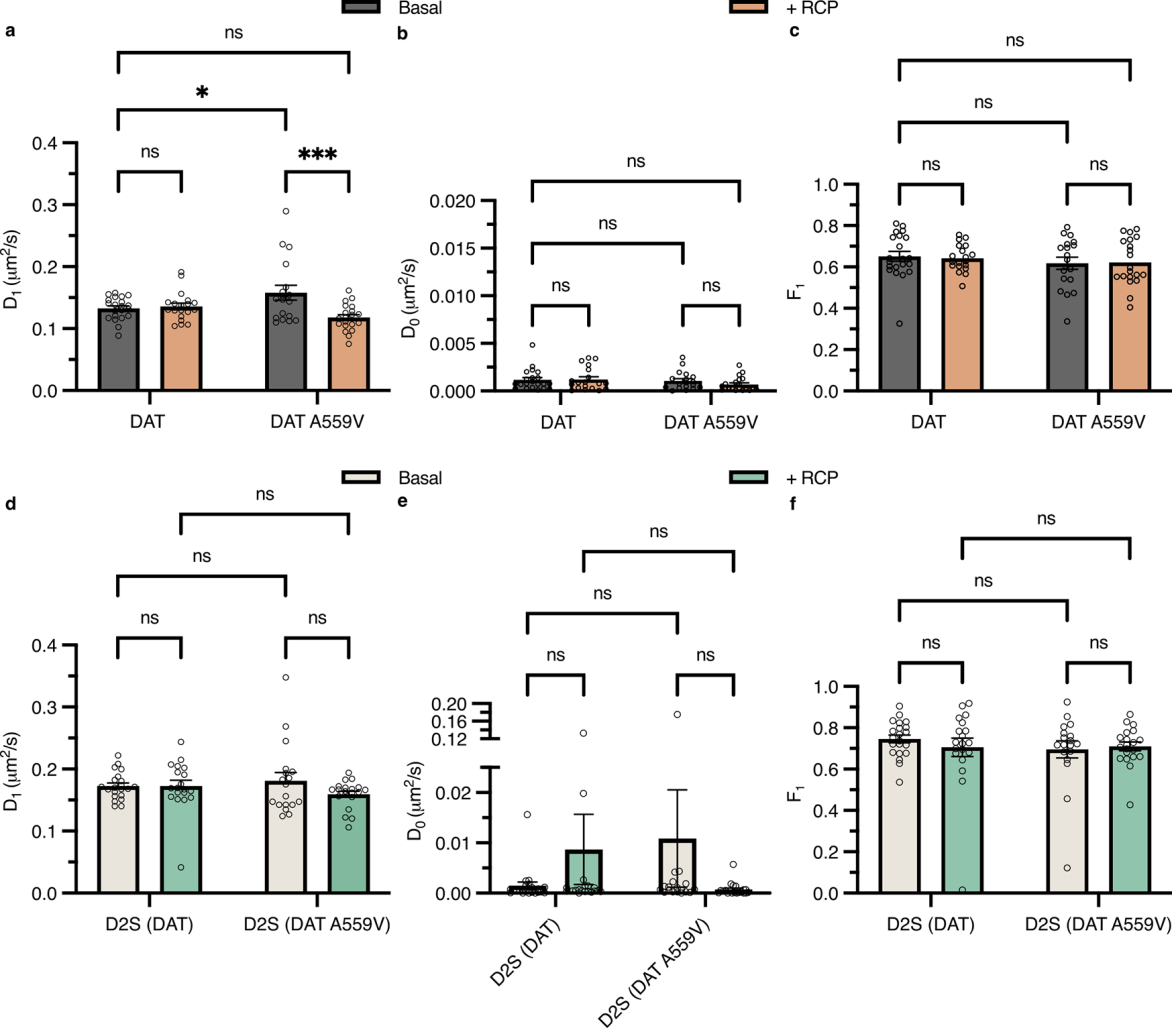
ExTrack global diffusion parameter fitting recapitulates single-color DAT and DAT A559V trends, but D2S diffusion is insensitive to RCP inhibition. (a–c) Two-state annotation model diffusion parameters generated per video acquisition for DAT or DAT A559V (DAT, *n* = 21; DAT + RCP, *n* = 19; DAT A559V, *n* = 18; DAT A559V + RCP, *n* = 20). (a) Diffusion coefficient for the mobile state 1. (b) Diffusion coefficient for the immobile state 0. (c) Fraction of particles in state 1. (d–f) diffusion parameters generated per video acquisition for D2S with its respective DAT or DAT A559V coexpressed (D2S (DAT), *n* = 21; D2S (DAT) + RCP, *n* = 19; D2S (DAT A559V), *n* = 18; D2S (DAT A559V) + RCP, *n* = 20). (d) Diffusion coefficient for the mobile state 1. (e) Diffusion coefficient for the immobile state 0. (f) Fraction of particles in state 1. (a–f) Two-way ANOVA with Holm-Sidak multiple comparisons test. Data are presented as mean ± SEM.

### RCP Inhibition Increases D2S Colocalization Lifetime with DAT and DAT A559V without Affecting the Total Number of Events

Due to the spatial limitations of fluorescence microscopy, we used DAT-D2S colocalization and diffusive state probability annotations as a proxy for dimer formation or membrane microdomain coresidence, where changes in the frequency or duration of colocalization-codiffusion events may support the presence of these phenomena. We analyzed the total number of colocalization events and the lifetimes of those events and compared whether DAT and DAT A559V differed under basal D2R blockade conditions. RCP treatment had no significant effect on the average number of colocalizations for DAT or DAT A559V (Figure 6a). However, RCP inhibition did increase the average colocalization lifetime (Figure 6c) and decreased the estimated dissociation rate (*k*_off_) (Figure 6d) for both DAT and DAT A559V. At basal the average lifetime and *k*_off_ for DAT-D2S (51 ± 0.7 ms and 25 ± 0.4 s^–1^) and DAT A559V-D2S (53 ± 0.8 ms and 26 ± 0.4 s^–1^) were the same. Under RCP inhibition, the average lifetime and *k*_off_ were significantly affected for DAT-D2S (60 ± 1 ms and 21 ± 0.3 s^–1^) and DAT A559V-D2S (60 ± 0.9 ms and 24 ± 1 s^–1^). These findings are the first lifetimes and *k*_off_ rates reported for DAT-D2S and are comparable to NMDA-D1 receptors^15^ and D2R dimers.^60^

**Figure 6:**
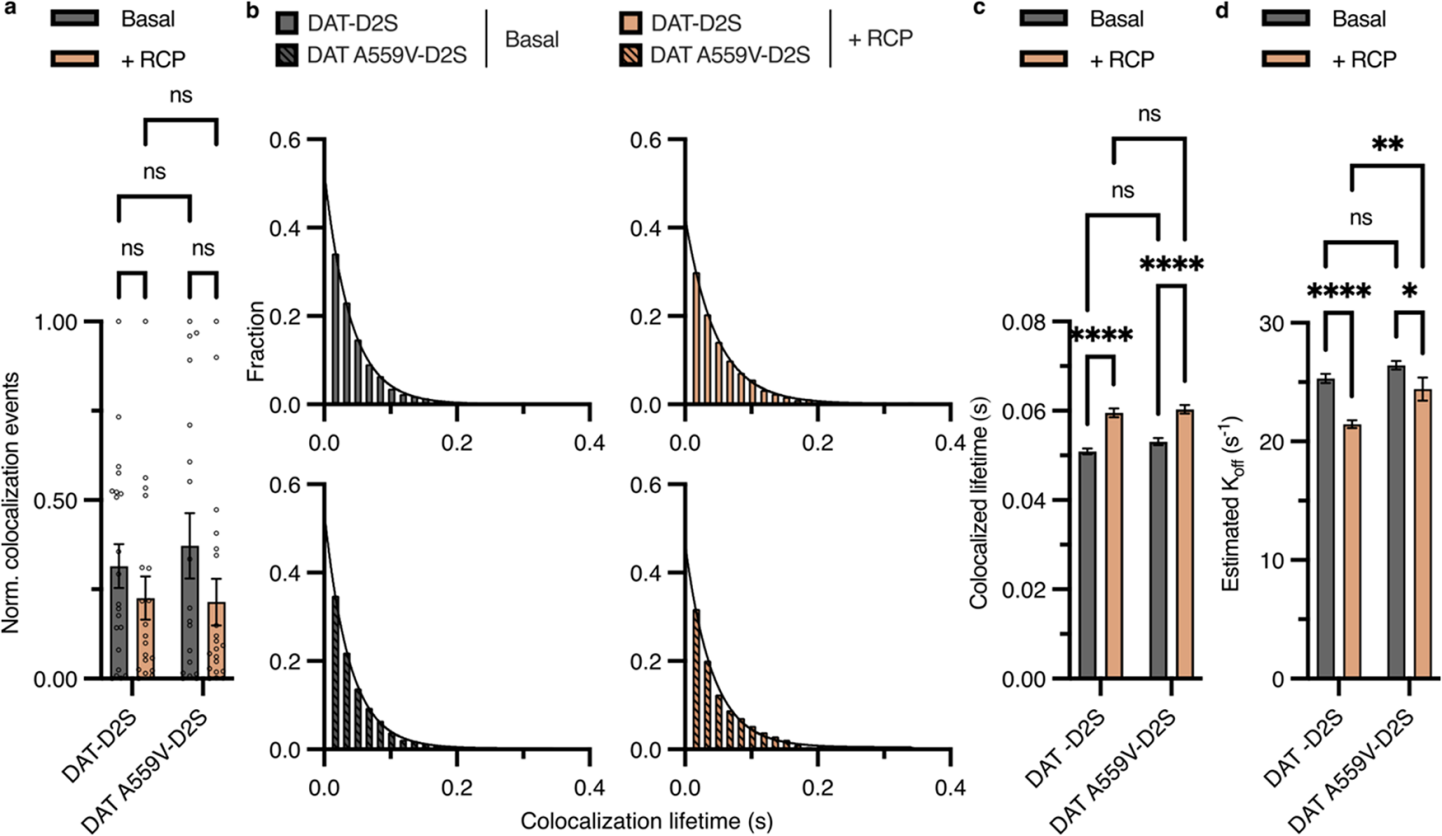
RCP inhibition increases colocalization lifetime with D2S for both DAT and DAT A559V without affecting the total number of colocalization events. (a) Normalized number of DAT-D2S colocalizing events for each video acquisition (DAT-D2S, *n* = 21; DAT-D2S + RCP, *n* = 19; DAT A559V-D2S, *n* = 18, DAT A559V-D2S + RCP, *n* = 20; Two-way ANOVA with Holm-Sidak multiple comparisons test). Data are presented as mean ± SEM. (b) Distribution and one exponential fit of colocalization lifetimes DAT-D2S, *n* = 4373; DAT-D2S + RCP, *n* = 2991; DAT A559V-D2S, *n* = 3759; DAT A559V-D2S + RCP, *n* = 3571. (c) Comparison of the average colocalization lifetime of panel b (Two-way ANOVA with Holm-Sidak multiple comparisons test). Data are presented as mean ± SEM. (d) Comparison of the estimated *k*_off_, i.e., dissociation rate (Two-way ANOVA with Holm-Sidak multiple comparisons test). Data are presented as mean ± SEM.

### DAT Diffusion State Probability Drops During Colocalization without Affecting D2S

We narrowed our colocalization analysis to uncover any changes in the diffusion state that may happen during DAT-D2S colocalization. Of the colocalizing trajectories, we further sorted the diffusion state probabilities, whether the points were before, during, or after colocalization. The greater the state probability, the greater the likelihood that any time point along a trajectory is in the mobile state. A probability threshold of 0.7, previously used by Simon et al.^54^ to assess the rebinding propensity of RodZ membrane proteins, was set for mobile state probabilities (Figure 7) in which median probabilities >0.7 are likely to be mobile and values <0.5 are more likely to be immobile. Under basal conditions, median transporter and receptor probabilities remained above 0.7 through the colocalizing trajectory (Figure 7a). However, under RCP inhibition, only DAT and DAT A559V fall below 0.7 during colocalization, while D2S was unchanged (Figure 7b). Moreover, DAT A559V’s state probability during colocalization fell to 0.5.

**Figure 7:**
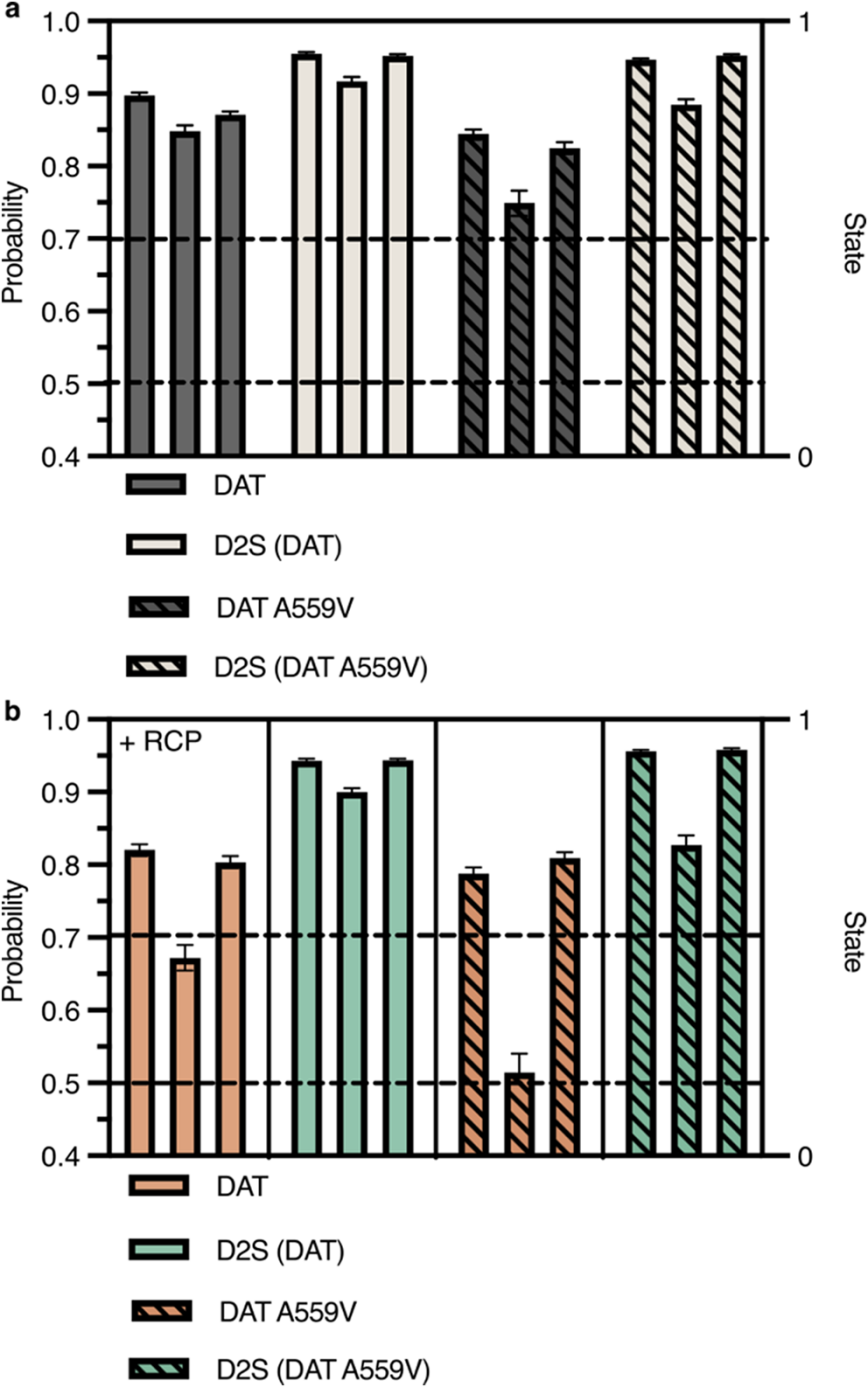
DAT and DAT A559V mobile state probability significantly decreases during D2S colocalization upon RCP inhibition. (a, b) Mobile state 1 probability for all colocalizing DAT and D2S trajectories nested as prior, during, and after colocalization. (a) Basal; (b) + RCP. Data are presented as median ± 95% CI.

## Discussion

Genetic variations that strongly impact DAT function contribute to the etiology of neuropsychiatric disorders such as attention-deficit hyperactivity disorder, bipolar disorder, and autism spectrum disorder, signifying DAT dysfunction as a major risk determinant for disease. Although the underlying molecular mechanisms of DAT disruption in behavioral disorders have not been fully elucidated, recent evidence suggests that mislocalization of neuropsychiatric disorder-associated DAT variants to membrane microdomains^29,39,61^ is a potential common culprit of DAT-mediated dopamine pathologies. Through dynamic imaging, we revealed the phenotypic rescue of the lateral diffusion rate of DAT A559V under D2R antagonism, whereas DAT diffusion was unaffected by D2R antagonism (Figure 2). Reports support endogenous expression of D2R in HEK-293 cells;^25,27^ however, it remains unclear as to which D2R isoform (D2S or D2L) is endogenously expressed. We were successful in capturing the dynamic effects of the D2R blockade on DAT lateral diffusion in this model. DAT and D2R may interact through physical associations, such as dimerization and functional interactions mediated by downstream signaling pathways. D2R activation influences DAT vertical trafficking by triggering intracellular signaling like extracellular signal-regulated kinase 1/2 (ERK1/2), PKCβ, and CaMKII, which regulate DAT phosphorylation, endocytosis, efflux, and recycling.^1,5,62–64^ These signaling events can result in changes to DAT surface expression, thereby modulating dopamine reuptake efficiency and synaptic levels, creating a bidirectional balance between receptor activity and transporter function.

Previously, we identified PKCβ-mediated regulation of DAT A559V diffusion, providing a critical link between N-terminal phosphorylation and DAT mobility.^29^ Here, we observed that the DAT A559V membrane mobility is insensitive to CaMKII-mediated regulation (Figure 3). Under basal conditions, DAT is minimally phosphorylated,^65–67^ whereas thorough pharmacological characterization indicates that DAT A559V’s N-terminus undergoes hyperphosphorylation at serine residues within amino acids 1–15. In this state, DAT A559V is resistant to amphetamine (AMPH) induced vertical trafficking^21^ and experiences ADE that is attenuated upon CaMKII or D2R inhibition.^25^ Notably, RCP not only inhibits ADE but also reduces elevated CaMKII autophosphorylation in DAT A559V expressing cells, further emphasizing the bidirectional relationship between D2R signaling and DAT regulation.^25^ Our diffusional data support the role of D2R in the post-translational regulation of DAT, whereby inhibiting D2R downstream signaling likely reduces DAT A559V N-terminal phosphorylation.^59,68^ RCP treatment may elicit a global effect by normalizing intracellular Ca^2+^ levels and, consequently, endogenous kinase activity. Reports do indicate DAT N-terminal serine phosphorylation by CaMKII, but DAT C-terminal threonine phosphorylation and distal C-terminal binding by CaMKII occur as well.^28,69,70^ While increased CaMKII phosphorylation may directly impact ADE, it is possible that CaMKII activity alone is insufficient to drive the abnormal diffusion for DAT A559V.

Co-immunoprecipitation studies have demonstrated a physical association between the DAT N-terminus (amino acids 1–15) and the D2R (both D2S and D2L) ICL3 (amino acids 311–344) that occurs independently of D2R stimulation, suggesting DAT/D2R may serve as a functional DAT unit,^30,71^ whereby dephosphorylation of the N-terminus could restore DAT-D2S dimers. To investigate this, we simultaneously imaged DAT/DAT A559V and D2S in HEK-293 cells and characterized instances of colocalization between the two proteins. Due to the exceptional brightness, photostability, and narrow emission of Qdots, we were able to achieve high frame rate (58 fps) imaging sensitive enough to uncover potential DAT-D2S transient interaction events. Our two-color data may challenge this notion, such that under basal conditions, DAT and D2S may not form stable dimers, as colocalization-codiffusion events could also reflect domain coconfinement rather than direct physical interaction (Figure 8). This interpretation is supported by global diffusion parameters, which revealed that while DAT A559V mobility mirrored previous findings (Figure 2), D2S mobility remained unaffected by transporter type or direct antagonism (Figure 5). Notably, under RCP inhibition, an increase in colocalization lifetime did not correspond to a decrease in D2S diffusion speed (*D*_1_), further supporting the absence of stable dimerization (Figure 6). Instead, D2S may reside in distinct membrane microdomains, with DAT transiently entering these domains without forming direct physical interactions. Lycas et al.^51^ reported limited nanodomain overlap between DAT and D2R under basal conditions in primary neurons, a finding consistent with our observations that DAT and D2R transiently coconfine rather than dimerize. This transient coconfinement could be regulated by DAT N-terminal phosphorylation (Figure 8), as suggested by Figure 7, where inhibiting all downstream kinase activity from D2S signaling reduced DAT and DAT A559V mobile state probabilities during colocalization to below 0.7, particularly for DAT A559V, which would experience a sharper decrease in N-terminal phosphorylation.

**Figure 8:**
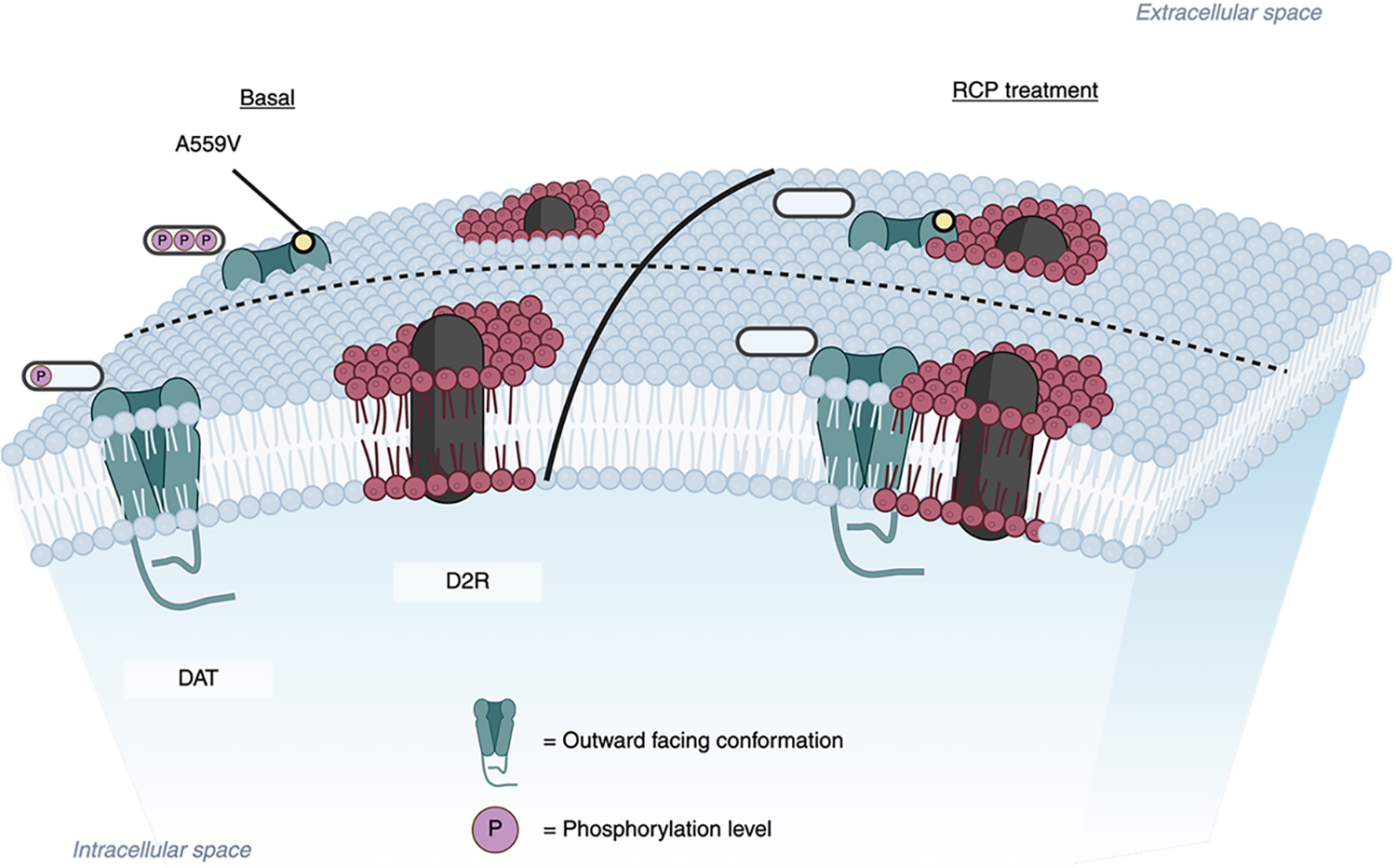
Cartoon model for DAT-D2S membrane microdomain coconfinement. The outward-facing conformation of DAT and DAT A559V transiently enters D2S membrane microdomains without forming a direct physical interaction with D2S, in which DAT N-terminal phosphorylation levels regulate the duration of microdomain coconfinement.

Two membrane surface conformational DAT pools exist, such that an inward-facing conformation (active site facing cytosol) is prone to clustering into nanodomains and an outward-facing conformation (active site facing extracellular space) is less prone to clustering into nanodomains, whereby D2R activation promotes DAT clustering and inhibition disperses DAT clustering.^51^ Prior studies demonstrated that D2R activation increases surface DAT, enhancing dopamine clearance,^72,73^ thus shifting DAT equilibrium toward the less mobile inward-facing conformation. Conversely, D2R inhibition reduces surface DAT levels, decreasing dopamine clearance and shifting DAT equilibrium toward the more mobile outward-facing conformation. Our DAT labeling approach selectively targets the outward-facing DAT conformation, sampling the more mobile population. This methodological distinction explains why we did not observe changes in DAT mobility upon D2R blockade. Our findings support that DAT A559V’s hyperphosphorylated N-terminus would make interactions with stabilizing membrane components, such as PIP2 and cholesterol,^74–76^ less favorable, resulting in increased mobility and reduced localization compared to wild-type DAT under basal conditions.

These results reveal the dynamic interplay between DAT and D2S, most importantly demonstrating that D2S inhibition can rescue DAT A559V aberrant mobility. Moreover, we tap into the potential of Qdot-based probes to maximize spatiotemporal resolution, providing the first documented report of a DAT-D2S colocalization lifetime in live cells. Our reported *k*_off_ rates are in excellent agreement with recently reported *k*_off_ rates between NMDA and D1 dopamine receptors and GluN1 subunit homodimers tracked using Qdots.^15^ Another report investigated D2R dimer lifetimes using molecular dyes, reporting lifetimes of 68 ms,^60^ further supporting our methodology. Future studies are needed to discern DAT mobility profiles between inward and outward-facing populations and how they interact with D2S. Additionally, to what extent does DAT A559V colocalize with membrane lipids such as cholesterol and/or PIP2? These data provide insight into how direct and indirect protein partner interactions impact the biophysical nature of DAT and could lead to novel therapeutic strategies that could replace or supplement classical dopamine signaling pathway targeting drugs.

## Materials and Methods

### Materials

Streptavidin Qdot 655 (SAv-Qdot 655), streptavidin Qdot 605 (SAv-Qdot 605), and F­(ab′)-goat antirabbit IgG (H + L) secondary antibody, Qdot 705 (Ab-Qdot 705) were purchased from Invitrogen. Rabbit anti-D2R (extracellular) antibody (ADR-002) was purchased from Alomone Laboratories. DMEM (Thermo Fisher), fetal bovine serum (Gibco), normal goat serum (Invitrogen), casein (Gibco), phosphate buffer saline (PBS, w/o Ca^2+^, Mg^2+^), and penicillin/streptomycin were purchased from Thermo Fisher. Raclopride (RCP), KN93, and KN92 were purchased from Milipore Sigma. Poly-d-lysine hydrobromide (mol wt 70,000–150,000) was purchased from Sigma-Aldrich. 35 mm uncoated No.1.5 coverslip-bottomed dishes were purchased from MatTek. No. 1.5 μ-Slide 4 Well Glass Bottom dishes were purchased from Ibidi.

### Coding Variant Constructs

pcDNA3.1­(+) hDAT and pcDNA3.1­(+) hDAT A559V are detailed.^77^ GFP-C1-CaMKIIalpha (Addgene plasmid no. 21226) and GFP-C1-CaMKIIalpha-K42R (Addgene plasmid no. 21221) were gifts from Tobias Meyer Ph. D. GFP-DRD2 (GFP-D2S; Addgene no. 24099) was a gift from Jean-Michel Arrang Ph. D. pcDNA3-EGFP (Addgene no. 13031) was a gift from Doug Golenbock, Ph.D. pCMV-XL5 D2S (NM_016574) was purchased from Origene.

### Cell Culture

HEK-293 cells were grown in a complete medium (DMEM and EMEM, respectively, with 2 mM glutamine, 10% fetal bovine serum (FBS), and 1% pen/strep) in a 37 °C incubator with 5% CO_2_. Cells were seeded in poly-d-lysine-coated (1 h at 37 °C) MatTek dishes at an appropriate density to obtain a subconfluent monolayer and grown for 24 h in the appropriate complete growth medium. For cells to be transiently transfected with one plasmid DNA, 500 ng of the appropriate DNA per MatTek dish using Lipofectamine 3000 according to the manufacturer’s instructions 24 h prior to labeling. For cells to be transiently transfected with two plasmid DNAs, 250 ng of each appropriate DNA per Ibidi well using Lipofectamine 3000 according to the manufacturer’s instructions 24 h prior to labeling.

### RCP, KN93, KN92, Treatments

Selective D2R antagonist RCP (1 μM) or CaMKII antagonist/inactive analogue KN93/KN92 (5 μM) were added directly to the transfected cells and incubated at 37 °C and 5% CO_2_ for 20 min prior to Qdot labeling.

### Qdot Labeling

Single-color Qdot labeling for DAT and A559V was employed by following a two-step protocol. After cells were allowed 24 h to achieve transporter or kinase expression, cells in 2 mL full growth medium were spiked with 20 μL 10 μM IDT444 suspended in PBS (w/o Ca^2+^, Mg^2+^) and incubated at 37 °C and 5% CO_2_ for 10 min. Three washes with warm DMEM Fluorobrite were performed prior to treating cells with 5 pM Qdot 2% dialyzed BSA in DMEM Fluorobrite. The Qdot-IDT444 DAT-labeled cells were washed three times with warm DMEM Fluorobrite.

Two-color Qdot labeling was employed by following a two-step protocol. After cells were allowed 24 h to achieve transporter and receptor expression, cells were washed once with blocking buffer (warm DMEM Fluorobrite with 5% goat serum and 1% casein) solution. Cells were incubated at 37 °C and 5% CO_2_ in premixed 5 μg/mL anti-D2S antibody in blocking buffer for 10 min (20 min if no RCP), spiked with RCP for 10 min, and then spiked with IDT444 for 10 min. Three washes with a warm blocking buffer were performed prior to treating cells with 100 nM Sav-Qdot 605 and Ab-Qdot 705 in blocking buffer. Three more washes with warm blocking buffer were performed.

### Microscopy

High-Speed *Spinning Disk Confocal Microscopy*. Time-series images were generated by an inverted Nikon-Ti Eclipse microscope system equipped with a Yokogawa CSU-X1 spinning disk confocal scanner unit, a heated stage, a 60× oil-immersion Plan Apo 1.4 NA objective, and a Photometrics Prime 95B scientific complementary metal-oxide-semiconductor (sCMOS) monochrome camera. Qdots were excited using a 405 nm solid-state diode laser (15 mW), and the emission was collected through a 641/75 emission filter. GFP and IDT307 molecules were excited using a 488 nm line (15 mW), and the emission was collected through a 525/36 emission filter. For SPT experiments, time-series were generated at 10 Hz for 1 min. *TIRF Microscopy.* Time-series images were generated by an ONI Nanoimager microscope system equipped with a heated stage, a 100× oil-immersion Plan Apo 1.45 NA objective, and a Hamamatsu Orca Flash4.0 v3 camera. Qdots (605, 655, or 705 nm emission), GFP, and IDT307 were excited using a 488 nm solid-state diode laser (250 mW), and the emission was passed through a dichroic mirror split at 640 nm (<20 nm channel overlap precision) and collected through 598/44 nm and 685/40 nm emission filters. Qdot 655 or 705 nm were used accordingly when GFP or IDT307 were present to mitigate spectral bleed through. All video data was gathered within 20 min of the final wash after Qdot labeling.

### Single Particle Tracking and Analysis

ImageJ TrackMate^52,53^ plug-in developed by Tinevez et al. was used to determine the center position of individual Qdots with subpixel accuracy and connect obtained Qdot coordinates into continuous trajectory segments.^78,79^ Individual Qdot positions were reconstructed into continuous segments using a maximum gap of 10 frames and a maximum displacement of 1 μm. Only trajectories of blinking Qdots (i.e., containing position gaps due to fluorescence intermittency) with a minimum duration of 50 frames were used for subsequent diffusion analysis to verify that single fluorophores were analyzed.

The diffusion coefficient *D*_MLE_ was determined by a maximum likelihood estimation (MLE) theoretical framework with a motion blur of 0.1.^80^ Trajectories of Qdots greater than the previously determined immobile particle threshold (5 × 10^–4^ μm^2^/s)^38,81^ were used to find MJD, MSD, and 5 s Displacement and for statistical comparison.

MJD values were calculated via

$$\mathrm{MJD}=\frac{1}{N}\sum_{i=1}^{N}d_{i}$$

where *d_i_* is the distance between the particle’s position at frame *i* and frame *i* + 1, and *N* is the total number of jumps measured.

MSD, ⟨*r* ^2^(*n*δ*t*)⟩, values were calculated for each of the trajectories collected for time intervals of 0.1–3.0 s in 0.1 s intervals via

$$\left\langle r^{2}\left(n\delta t\right)\right\rangle =\frac{1}{N-n}\sum_{j=0}^{N-n-1}\{{\left[x\left(j\delta t+n\delta t\right)-x\left(j\delta t\right)\right]}^{2}+{\left[y\left(j\delta t+n\delta t\right)-y\left(j\delta t\right)\right]}^{2}\}\left(n=0,1,2,...,N-1\right)$$

where δ*t* is the temporal resolution, (*x*(*j*δ*t*), *y*(*j*δ*t*)) is the coordinate at *t* = *j*δ*t*, and *N* is the number of total frames recorded during a single trajectory. The diffusion coefficient *D*_MLE_ was determined by a maximum likelihood estimation (MLE) theoretical framework with a motion blur of 0.1 and MSD.^80^ Trajectories of Qdots greater than the previously determined immobile particle threshold 5 × 10^–4^ μm^2^/s^38,81^ were used for statistical comparison. *For single-color tracking*, the localization accuracy of the central position of the Qdot in our imaging approach was estimated to be ∼20 nm based on 2411 Qdot trajectories immobilized onto a coverslip.

*For two-color tracking*, TrackMate trajectory files were exported into the ExTrack^54^ python package and the default arguments were used to give equal weight to all diffusing particles. ExTrack is an MLE method based on a Hidden Markov Model (HMM) that approximates a continuous-time process by a discrete-time Markov model^82^ to generate global diffusion parameters,^83^ diffusion state probabilities, and refined trajectory positions for each transporter and receptor. A computational algorithm written in MATLAB was developed to extract distances separating DAT and D2S from refined trajectory positions generated from ExTrack. DAT and D2S were considered colocalizing when their Euclidean distance fell within the threshold (1 pixel; 0.117 μm) at the same time step. This threshold was derived from the acquisition parameters used for TIRF microscopy. Colocalizing DAT and D2S trajectories were further annotated as being *before, during*, or *after* colocalization, and the corresponding state probability was stored. The localization accuracy of the central position of Qdot 605 and 705 in our imaging approach was estimated to be ∼9 and ∼16 nm, respectively, based on 7381 trajectories for each Qdot.

### Statistical Analysis

No statistical methods were used to predetermine the sample size. The sample size was based on previous publications with similar models and experiments. To ensure reproducibility, all results were derived from at least three biological and technical replicates. All statistical tests were performed using GraphPad Prism. Test details and statistical outcomes are reported in the relevant figures and figure legends.

## Supplementary Material



## Data Availability

Data, resources, and codes used are available from the corresponding author upon request.

## Notes

SPT data for DAT A559V under endogenous CaMKII inhibition (Figure S1), anti-D2S antibody specificity for D2S verification (Figure S2), SPT data for DAT A559V coexpressed with GFP-D2S or EGFP (Figure S3), Qdot blinking behavior for both Qdots used for two-color tracking (Figure S4), and ExTrack diffusion parameters for Qdots adhered to glass coverslip (Figure S5) (PDF)
