# Supplementary material for: Multicolor Quantum Dot Tracking Uncovers Phenotypic Rescue of DAT A559V Aberrant Diffusion Upon D2R Antagonism
Source: ACS Chem Neurosci. 2026 Feb 5;17(4):791–804. doi: 10.1021/acschemneuro.5c00897 (PMC12921695; doi:10.1021/acschemneuro.5c00897)
Supplement: Supplementary file 1 [file cn5c00897_si_001.pdf]

Supporting Information

Multicolor quantum dot tracking uncovers phenotypic rescue of DAT A559V aberrant diffusion upon D2R antagonism

Ruben Torres[1,2,3], Oleg Kovtun[1], James R. McBride[1,3,4], Laurel G. Bellocchio[1,2,3], and Sandra J. Rosenthal[1,2,3,5,6,7]*

[1]Department of Chemistry, Vanderbilt University, Nashville, TN 37240, USA

[2]Vanderbilt Institute of Chemical Biology, Vanderbilt University, Nashville, TN37240, USA

[3]Vanderbilt Institute for Nanoscale Science and Engineering, Vanderbilt University, Nashville 37240, TN, USA

[4]Department of Electrical and Computer Engineering, Vanderbilt University, Nashville, TN 37240, USA

[5]Department of Pharmacology, Vanderbilt University, Nashville, TN 37240, USA

[6]Department of Chemical and Biomolecular Engineering, Vanderbilt University, Nashville, TN 37240, USA

[7]Vanderbilt Interdisciplinary Materials Science Program, Vanderbilt University, Nashville, TN 37240, USA

*Corresponding Author Email: sandra.j.rosenthal@Vanderbilt.edu

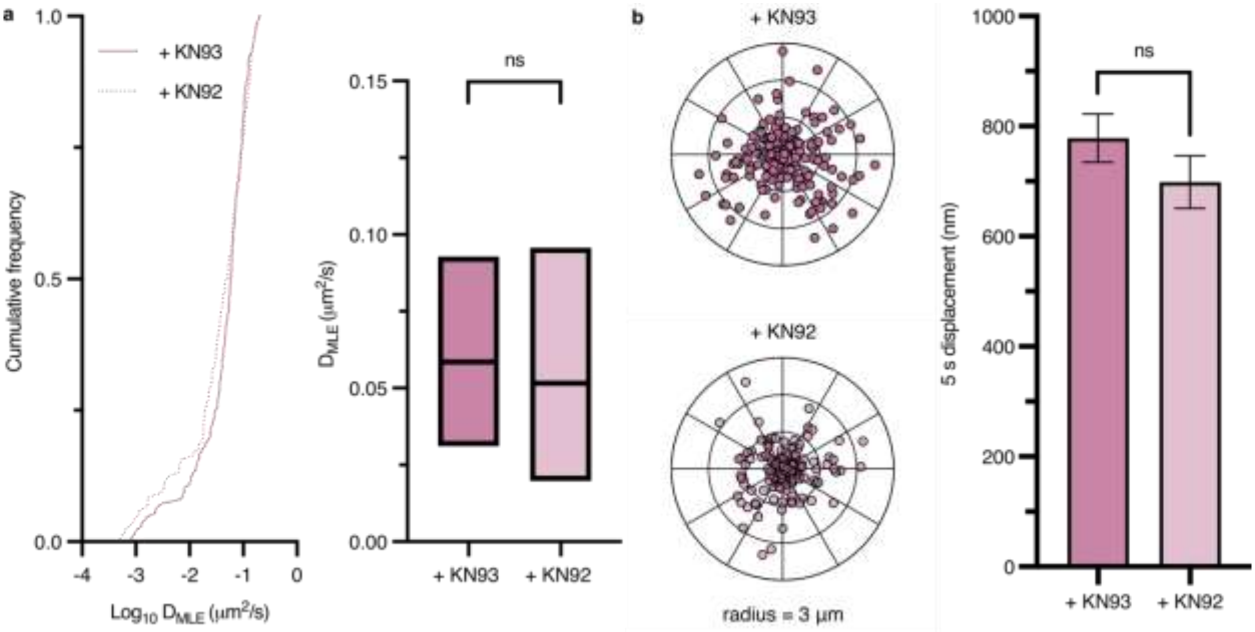

Figure S1: DAT A559V mobility is insensitive to endogenous CaMKII inhibition. a Cumulative frequency distribution of diffusion coefficients (D_{MLE}) for DAT A559V + KN93 or + KN92 treatment (Kolmogorov-Smirnov 2-sample test, $p > 0.05$) **b** Box plot showing the diffusion coefficient distributions in panel **a**. The median value is shown as the colored horizontal line in the box, the 25-75% IQR interval corresponds to the length of the colored box (Kolmogorov-Smirnov 2-sample test). **c** Polar plots (*left*; outer radius limit = 3 μm) and 5 s displacement bar graph (*right*; two-tailed unpaired t test). Displacements are normalized to their spatial origin. Data are presented as mean +/- SEM. *N* trajectories (+ KN93, *n* = 221; + KN92 *n* = 153).

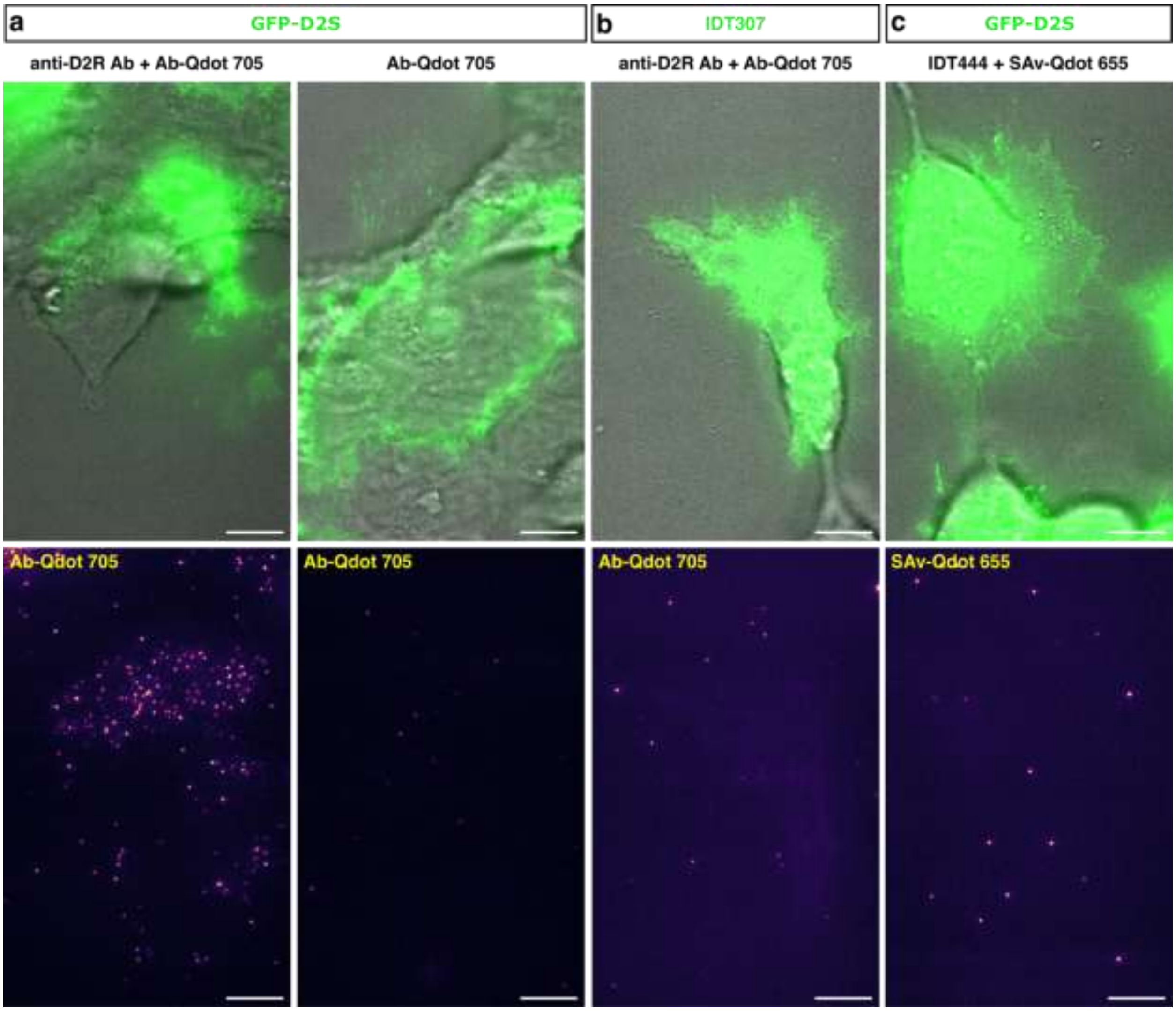

Figure S2: Verification of anti-D2S antibody in transiently transfect HEK-293 cells. **a** Representative images of cells expressing GFP-D2S and treated with (*left*) or without (*right*) primary anti-D2S antibody prior to secondary Ab-Qdot 705 and the corresponding Ab-Qdot 705 signal below (*n* = 4 cells). **b** Representative image of cells expressing DAT, labeled with IDT307 to confirm DAT expressing cells and treated with primary anti-D2S antibody and secondary Ab-Qdot 705 (*n* = 5 cells). **c** Representative image of cells expressing GFP-D2S treated with DAT-specific labeling scheme **from Fig. 1** to demonstrate labeling specificity for DAT and D2S (*n* = 4 cells).

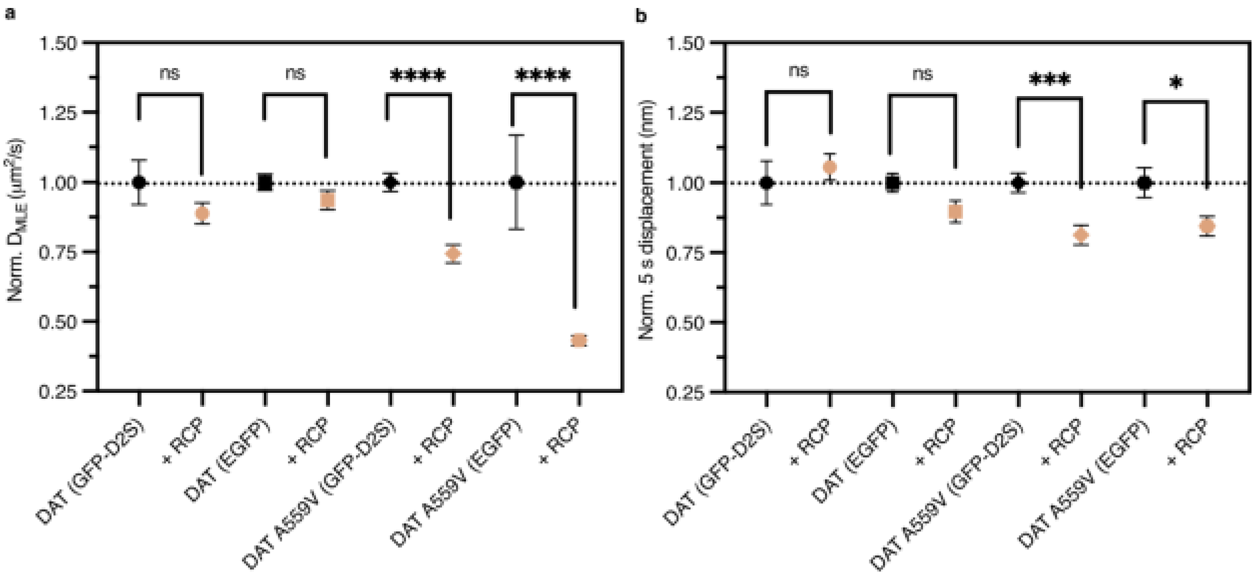

Figure S3: DAT A559V diffusion recovery upon RCP inhibition is conserved in transiently transfected HEK-293 cells co-expressing GFP-D2S or EGFP. a Normalized diffusion coefficients (D_{MLE}) and **b** 5 s displacements of DAT or DAT A559V co-expressed with GFP-D2S or EGFP (control). Data for RCP-treated groups are normalized to their respective basal condition (**a**, Mann-Whitney test; **b**, two-tailed unpaired t test). Data are presented as mean +/- SEM. *N* trajectories (DAT (GFP-D2S), *n* = 202; DAT (GFP-D2S) + RCP, *n* = 513; DAT (EGFP), *n* = 631; DAT (EGFP) + RCP *n* = 383; DAT A559V (GFP-D2S), *n* = 753; DAT A559V (GFP-D2S) + RCP, *n* = 511; DAT A559V (EGFP), *n* = 480; DAT A559V (EGFP) + RCP, *n* = 554).

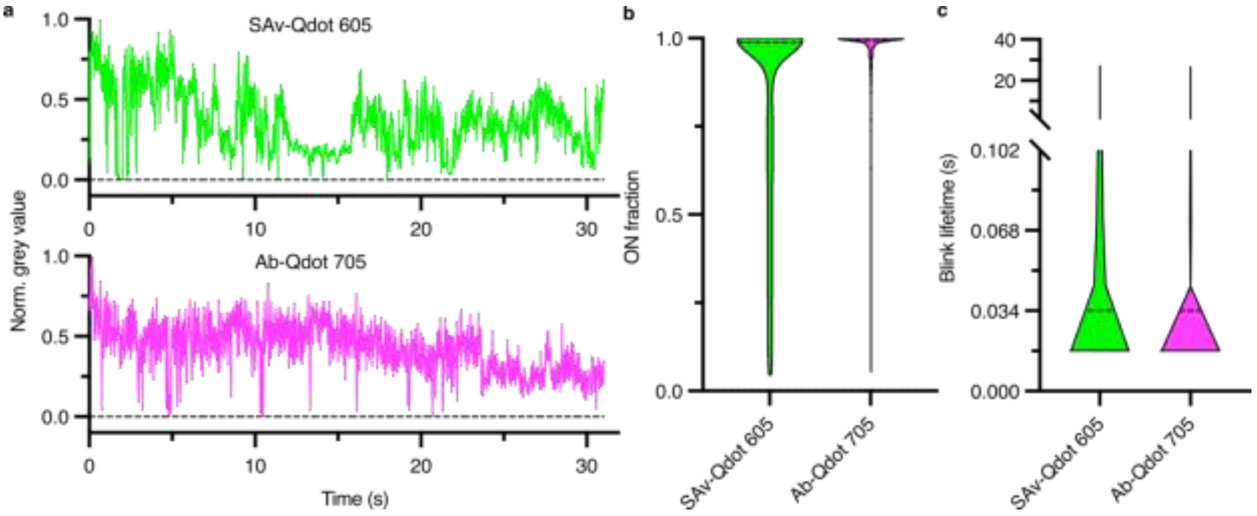

Figure S4: Blinking behavior of SAv-Qdot 605 and Ab-Qdot 705 using for two-color imaging. **a** Representative intensity transients of SAv-Qdot 605 (*top*) and AB-Qdot 705 (*bottom*). Instances where intensity minima hit background noise baseline (0) are considered blinking. **b**. Violin plot of ON-fraction populations – the fraction of time a Qdot spends in the emissive state over the course of video acquisition – and **c**. blinking durations for both SAv-Qdot 605 (n = 618) and Ab-Qdot 705 (n = 813). The median value is shown as the dotted horizontal line.

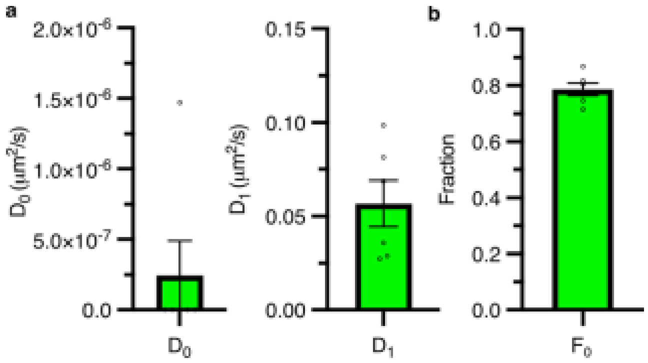

Figure S5: Two-state model diffusion parameters for SAv-Qdot 605 adhered to glass coverslip a Diffusion coefficient for state 0 (*left*) and state 1 (*right*). **b** Fraction of particles in state 0 (*n* = 6 videos).
